# Resonant Spin Amplification and Accumulation in MAPbI_3_ Single Crystals

**DOI:** 10.1002/advs.202502735

**Published:** 2025-05-07

**Authors:** Erik Kirstein, Dmitri R. Yakovlev, Evgeny A. Zhukov, Nataliia E. Kopteva, Bekir Turedi, Maksym V. Kovalenko, Manfred Bayer

**Affiliations:** ^1^ Technische Universität Dortmund Experimentelle Physik 2 44227 Dortmund Germany; ^2^ Department of Chemistry and Applied Biosciences Laboratory of Inorganic Chemistry, ETH Zürich Zürich 8093 Switzerland; ^3^ Department of Advanced Materials and Surfaces Laboratory for Thin Films and Photovoltaics Empa ‐ Swiss Federal Laboratories for Materials Science and Technology Dübendorf 8600 Switzerland; ^4^ Research Center FEMS Technische Universität Dortmund 44227 Dortmund Germany

**Keywords:** carrier spin dynamics, lead halide perovskite crystals, resonant spin amplification, spintronics, time‐resolved kerr ellipticity

## Abstract

Quantum technologic and spintronic applications require reliable semiconducting materials that enable a significant, long‐living spin polarization of electronic excitations and offer the ability to manipulate it optically in an external field. Due to the specifics of band structure and remarkable spin‐dependent properties, the lead halide perovskite semiconductors are suitable candidates for that. Here, the carrier spin dynamics in a MAPbI_3_ (MA = methylammonium) perovskite single crystal with thickness of 20 µm are studied by the time‐resolved Kerr ellipticity technique at cryogenic temperatures. Long times of longitudinal electron spin relaxation *T*_1_ = 30 ns and transverse electron spin dephasing $T_{2,e}^{*}=21$ ns are found. The spin dynamics lasting longer than the applied laser pulse repetition period give rise to spin accumulation effects. They are exploited through the resonant spin amplification, polarization recovery, and spin inertia techniques to study the electron and hole spin systems coupled with the nuclear spins. These results establish the lead halide perovskite semiconductors as suitable platform for quantum technologies relying on spin‐dependent phenomena.

## Graphical abstract

Carrier spin dynamics are studied in a 20 µm thin MAPbI_3_ perovskite single crystal using time‐resolved Kerr ellipticity at cryogenic temperatures. Long spin relaxation (30 ns) and dephasing (21 ns) enable spin accumulation. Spin accumulation effects are discussed in detail along with carrier‐nuclear spin interactions.

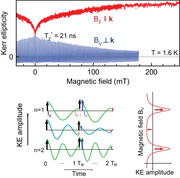

## 1 Introduction

Lead halide perovskite semiconductors have evolved toward a versatile platform for photovoltaic and optoelectronic applications.^[^ ^1^, ^2^, ^3^ ^]^ Their spin‐dependent properties are promising for spintronic and spin‐orbitronic applications.^[^ ^1^, ^4^, ^5^, ^6^ ^]^ Band structure and crystal symmetry of the lead halide perovskites differ considerably from that of conventional III‐V and II‐VI semiconductors with zinc blende structure, establishing them as novel material platform for the spin physics of semiconductors.^[^ ^7^, ^8^ ^]^ Spin‐dependent parameters, like the Landé *g*‐factor, and spin relaxation mechanisms of electrons, holes and excitons, as well as nuclear spin dynamics provide access to the band parameters and their anisotropy, which often cannot be measured by other techniques. In turn, knowledge of these parameters, combined with flexibility of synthesis of the lead halide perovskites with band gaps tunable across the whole visible spectral range from 1.5 to 3.2 eV, allows tailoring of the spin‐dependent properties.

Time‐resolved Faraday/Kerr rotation are widely used magneto‐optical techniques to study the spin dynamics in semiconductor crystals and their nanostructures.^[^ ^9^, ^10^ ^]^ They exploit circularly polarized pump pulses to generate spin‐oriented electrons and holes, which spin dynamics are detected by linearly polarized probe pulses via their Faraday or Kerr rotation. The techniques have been successfully used to investigate the coherent spin dynamics of electrons and holes in lead halide perovskite semiconductors and to measure *g*‐factors, spin relaxation and spin dephasing times. Their power has been demonstrated for bulk crystals of CsPbBr_3_,^[^ ^11^, ^12^ ^]^ FA_0.9_Cs_0.1_PbI_2.8_Br_0.2_,^[^ ^13^ ^]^ MAPbI_3_,^[^ ^14^, ^15^ ^]^ FAPbBr_3_,^[^ ^16^ ^]^ MAPbBr_3_,^[^ ^17^ ^]^ and polycrystalline films of CsPbBr_3_,^[^ ^18^, ^19^ ^]^ MAPb(Cl,I)_3_,^[^ ^20^ ^]^ MAPbI_3_,^[^ ^21^, ^22^ ^]^ and FAPbI_3_^[^ ^23^ ^]^ (FA = formamidinium). Universal dependences of the electron and hole *g*‐factors on the band gap were found for these materials.^[^ ^7^, ^24^ ^]^ A collection of times characterizing the spin dynamics can be found in ref. [16]. Commonly, the spin dephasing times are longer in single crystals, where the longest time for electrons of 11.5 ns was reported for FAPbBr_3_^[^ ^16^ ^]^ and for holes of 8 ns for MAPbBr_3_.^[^ ^17^ ^]^ In films, these times often are shorter than 1 ns, while recently comparably long times were reported for MAPbI_3_ films:^[^ ^21^, ^22^ ^]^ 4.4 ns for electrons and 7 ns for holes. For comparison, the longest times in MAPbI_3_ single crystals are 11 ns for electrons and 6 ns for holes.^[^ ^15^ ^]^

For the spin dynamics time scales approaching or exceeding the repetition period of the exciting pump laser pulses, spin accumulation can arise from the addition of spin dynamics induced by consecutive pulses. In order to gain comprehensive information on spin relaxation times in this case, not only measuring the spin dynamics, but also measuring the spin signals at a fixed pump‐probe delay while scanning the magnetic field is instructive. Three such techniques exploiting the application of an external magnetic field have been developed and successfully used for conventional III‐V and II‐VI semiconductors: resonant spin amplification (RSA),^[^ ^25^, ^26^, ^27^ ^]^ spin mode‐locking,^[^ ^10^, ^28^ ^]^ and spin inertia.^[^ ^29^ ^]^ For lead halide perovskite semiconductors, the RSA method has not been reported so far. The spin mode‐locking has the same origin of spin synchronization as the RSA effect, but appears in systems with a large dispersion of *g*‐factors within an ensemble of carrier spins.^[^ ^30^ ^]^ It was found for holes confined in CsPb(Br,Cl)_3_ nanocrystals.^[^ ^31^ ^]^ The spin inertia method was used for measuring the longitudinal spin relaxation times of carriers in CsPbBr_3_,^[^ ^11^ ^]^ MAPbI_3_,^[^ ^14^ ^]^ and FAPbBr_3_.^[^ ^16^ ^]^

In this paper, we use these techniques to study the spin dynamics of charge carriers in MAPbI_3_ single crystals at low temperatures in the orthorhombic phase. We find very long spin dephasing times reaching 21 ns for electrons, which allow us to enter the spin accumulation regime and use it for obtaining comprehensive information about the spin properties. Also the influence of the nuclear spins interacting with the carrier spins for the spin dynamics can be investigated by means of the polarization recovery (PR) effect and dynamic nuclear polarization (DNP).

## 2 Results

### 2.1 Optical Properties

**Figure 1:**
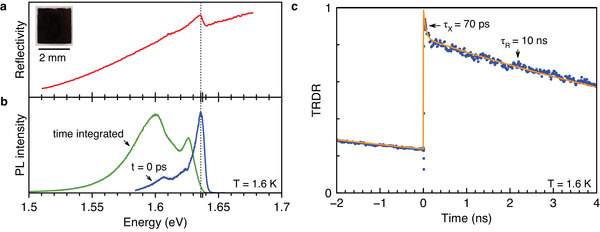
Optical properties of MAPbI_3_ single crystal at *T* = 1.6 K. a) Reflectivity spectrum showing a dispersively shaped resonance, with the exciton energy at 1.636 eV marked by the dashed line. The inset gives a representative picture of the sample, adapted from ref. [39]. b) Photoluminescence measured for pulsed excitation right after the excitation pulse at *t* = 0 ps (blue) and time integrated (green). c) Time‐resolved differential reflectivity (TRDR). Blue dots are the experimental data and orange line is a biexponential fit with the shorter decay time $\tau_{X}=70$ ps, provided by the exciton recombination, and the longer decay time $\tau_{R}=10$ ns, associated with recombination of spatially separated electrons and holes. The power densities of pump and probe are 1 W cm^−2^.

The sample under study is a crystal of hybrid organic–inorganic perovskite MAPbI_3_ with a thickness of 20 µm. In all the presented experiments, the light **k**‐vector is perpendicular to the sample surface, i.e., aligned along the z‐axis, which coincides with the normal to the sample surface. The optical properties of the MAPbI_3_ crystal at *T* = 1.6 K are presented in **Figure** **1**. In the reflectivity spectrum shown in Figure 1a a pronounced exciton resonance is observed at *E*_X_ = 1.636 eV. The exciton binding energy in bulk MAPbI_3_ is 16 meV,^[^ ^32^ ^]^ and the estimated bandgap energy is *E*_g_ = 1.652 eV.

Time‐resolved and time‐integrated photoluminescence (PL) spectra measured with a streak‐camera are shown in Figure 1b. Right after the laser excitation, the PL maximum is located at 1.636 eV energy, corresponding to the emission of excitons, see blue spectrum. At this energy the signal shows a multi‐exponential decay with two fast components characterized by times of 15 and 85 ps, corresponding to the exciton dynamics, and a much longer one with characteristic time of 520 ps due to recombination of spatially‐separated localized electrons and holes, see ref. [33] for details. It is a common feature of bulk lead halide perovskite semiconductors that the emission lines from recombination of electron‐hole pairs and of excitons are spectrally overlapping.^[^ ^8^, ^14^, ^34^, ^35^, ^36^ ^]^ Their contributions, however, can be separated by time‐resolved techniques and also by polarized PL in magneto‐optical experiments.^[^ ^34^ ^]^ In the time‐integrated PL spectrum (green line in Figure 1b) the PL maximum is shifted toward lower energies down to 1.627 eV. Also the low energy band with a maximum at 1.600 eV becomes most intense here.

We measure also the population dynamics of excitons and carriers by time‐resolved differential reflectivity, see Figure 1c. The dynamic evolution shows bi‐exponential behavior. The short component with τ_X_ = 70 ps decay time can be associated with the exciton lifetime. The long‐lived component has a larger amplitude and a decay time of τ_R_ = 10 ns. As a result, it does not fully decay during the repetition period between the laser pulses of *T*_R_ = 13.2 ns so that a finite intensity is seen at negative delays. Note, that in this experiment we use linearly polarized light for the pump and probe beams and, therefore, measure the population dynamics which is insensitive to spin polarization.

Note that recently we found in a similar, thin MAPbI_3_ crystals optical spin orientation of excitons with a very high degree of 85%, detected via polarized photoluminescence,^[^ ^33^, ^37^ ^]^ which is robust against energy detuning of the excitation laser from the exciton resonance. It represents a common property of bulk lead halide perovskite semiconductors irrespective of their crystal symmetry (about cubic, tetragonal, orthorhombic)^[^ ^37^ ^]^ and evidencing the absence of the Dyakonov–Perel spin relaxation mechanism.

### 2.2 Spin Dynamics of Electrons and Holes

We use the time‐resolved Kerr ellipticity (KE) technique to study the spin dynamics of electrons and hole. The spin polarization of electrons and holes is induced by circularly polarized pump pulses. According to the optical selection rules for lead halide perovskite semiconductors,^[^ ^8^ ^]^ a σ^+^ polarized photon with angular momentum +1 generates a pair of an electron and a hole both having spin +1/2. Vice verse, a σ^−^ polarized photon generates carriers with spin −1/2. The induced carrier spin polarization is detected via the Kerr rotation effect experienced by linearly polarized probe pulses, which are delayed in time relative to the pump pulses. For technical reasons, we measure in our experiments not directly the Kerr rotation, but the Kerr ellipticity, i.e., the presence of a small circularly polarized component in the reflected probe light, as it has maximum intensity at the exciton resonance.^[^ ^38^ ^]^

**Figure 2:**
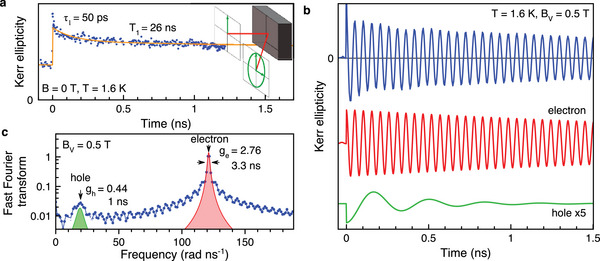
Spin dynamics in the MAPbI_3_ single crystal measured at 1.637 eV. a) Spin dynamics at zero magnetic field (blue dots) and corresponding biexponential fit (orange line). The fit gives the spin lifetime τ_1_ = 50 ps and the spin relaxation time *T*_1_ = 26 ns. Note that *T*_1_ exceeds the laser pulse repetition rate $T_{R}=13.2$ ns, but due to the implemented experimental method of double modulation, the signal is nearly offset‐free and the signal at negative time delays can be included in the fit. Sketch illustrating the Kerr ellipticity effect, converting the linearly polarized probe beam into an elliptical one. b) Spin dynamics in a magnetic field applied in the Voigt geometry: *B*_V_ = 0.5 T. *T* = 1.6 K, pump power of 1.6 W cm^−2^. Below the vertically shifted electron and hole dynamics, decomposed by simulating the experimental dynamics, are shown. c) Fast Fourier transform (FFT) (blue line) of the signal shown in panel (b). The two Lorentzian fit curves give the electron and hole contributions. The denoted parameters are discussed in the text.

The KE dynamics measured at zero magnetic field for 1.637 eV photon energy, corresponding to the exciton resonance, are shown in **Figure** **2**a. The spin dynamics show two components, accordingly fitted with a biexponential decay functions. The short decay time of τ_1_ = 50 ps is close to the exciton recombination time τ_X_. Therefore, it can be assigned to the lifetime of the exciton spin $T_{\text{s,X}}^{-1}=\tau_{\text{s,X}}^{-1}+\tau_{X}^{-1}$, which is limited by exciton recombination. Here τ_s,X_ characterizes the exciton spin relaxation. Note that our studies of the exciton optical orientation effect and the magnetic‐field‐induced exciton circular polarization in similar MAPbI_3_ crystals confirm that we are in a regime where τ_s,X_ ≫ τ_X_ holds at *T* = 1.6 K for them.^[^ ^33^ ^]^

The long‐lived component in the KE dynamics of Figure 2a has a decay time of *T*_1_ = 26 ns. Accordingly, we assign it to the longitudinal spin relaxation time of resident electrons and/or holes. Note that the *T*_1_ time greatly exceeds the times involved in the exciton dynamics, confirming our interpretation of the long‐living dynamics arising from spatially‐separated resident carriers.

In a magnetic field applied in the Voigt geometry (**B**_V_⊥**k**), i.e., perpendicular to the induced spin polarization, the carrier polarization undergoes Larmor spin precession about the field direction.^[^ ^10^ ^]^ This results in oscillations in the KE dynamics, shown by the blue line in Figure 2b for *B*_V_ = 0.5 T. The dynamics comprise two components, whose contributions we separate by a fit with Equation (16) and show in the same figure: the faster oscillating component with the Larmor precession frequency ω_L,e_ = 121.18 rad ns^−1^ and the spin dephasing time $T_{2,e}^{*}=3.3$ ns belong to the electrons, while the slower oscillating one (ω_L,h_ = 19.23 rad ns^−1^ and $T_{2,h}^{*}=0.35$ ns) arise from the holes. This identification of the components is done on basis of the known electron and hole *g*‐factors for MAPbI_3_ crystals, which are anisotropic and are in the ranges of *g*_e_ = +2.46 to +2.98 and *g*_h_ = −0.28 to −0.71.^[^ ^7^ ^]^

The Larmor precession frequency ω_L_ is proportional to the Zeeman splitting *E*_Z_ which depends on the Landé *g*‐factor according to:

(1) $$E_{\text{Z,e(h)}}=\hbar \omega_{\text{L,e(h)}}=\left|g_{\text{e(h)}}\right|\mu_{B}B\;,$$

with μ_B_ being the Bohr magneton. From the data presented in Figure 2b, we evaluate |*g*_e_| = 2.76 ± 0.10 and |*g*_h_| = 0.44 ± 0.10. Note that the *g*‐factor signs cannot be identified from these experiments, but were determined for bulk MAPbI_3_ using the dynamic nuclear polarization effect.^[^ ^13^ ^]^

Two frequencies, corresponding to the two oscillating components in the KE dynamics, are also well seen in the Fast Fourier Transform (FFT) spectrum (Figure 2c). Here, the two peaks correspond to the hole and electron Larmor precession frequencies, and their full widths at half maximum (FWHM) give an estimate of their respective spin dephasing times: Δω_e_ = 1.91 rad ns^−1^ ($T_{2,e}^{*}=2\pi /\Delta \omega_{e}=3.3$ ns) and Δω_h_ = 6.03 rad ns^−1^ ($T_{2,h}^{*}=2\pi /\Delta \omega_{h}=1$ ns).

Note the the amplitude of the hole spin contribution is much smaller than that of the electron, which might originate from a sample‐specific drain of resident holes, see.^[^ ^39^ ^]^ Commonly, comparable amplitudes for the electron and hole spin signals are observed in bulk lead halide perovskites.^[^ ^11^, ^13^ ^]^

**Figure 3:**
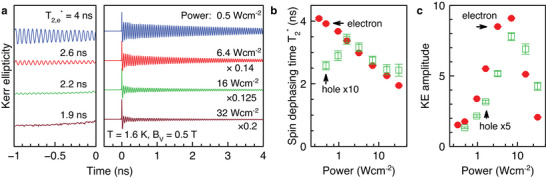
Spin dynamics at various pump powers, measured at *B*_V_ = 0.5 T. a) KE dynamics for a set of different pump powers, but constant probe power of 0.3 W cm^−2^. The sample is immersed in superfluid helium at *T* = 1.6 K. Most prominent at negative time delays (for the actual time delay, add *T*_R_ = 13.2 ns), the observed long‐living spin oscillations vanish with increasing pump power. The signal amplitudes in the left panel are magnified by a factor of 40. b) Pump power dependence of the spin dephasing times $T_{2}^{*}$ of electrons and holes. The hole times are multiplied by a factor of 10 for better visibility. c) Pump power dependence of the KE amplitudes. The hole amplitude is magnified by a factor of 5. The data in (b,c) are gathered via fits of the data set shown in (a) with Equation (16).

In **Figure** **3**a, we show the KE dynamics measured at pump powers varied from 0.5 W cm^−2^ to 32 W cm^−2^. The dynamics' amplitudes are normalized. One sees that with increasing power the electron spin dephasing becomes faster, as seen in a pronounced manner from the decreasing oscillation amplitude at negative delays, which corresponds to a positive delay of about *T*_R_ = 13.2 ns. One can see in Figure 3b that $T_{2,e}^{*}$ shortens from 4 ns to 2 ns. The hole spin dephasing time is much shorter, being about 200 ps, and weakly depends on pump power.

The KE amplitudes increase with the power growing up to about 6.4 W cm^−2^ and then drop with a further power increase to 32 W cm^−2^ (Figure 3c). We explain the decrease by electron heating and delocalization. In general, the carrier coherent spin dynamics are very sensitive to the bath temperature, as we will show for the studied sample below in Section 2.7. An increased carrier temperature is equivalent to an overall increased sample temperature.

Most notably, in Figure 3a at negative time delays and for small pump power densities, the spin precession persists reflecting spin accumulation over several pump periods. We will exploit this accumulation effect in the next section.

### 2.3 Resonant Spin Amplification

Resonant spin amplification (RSA) has been found in n‐type doped GaAs^[^ ^25^ ^]^ and extended experimentally and theoretically to many conventional III‐V and II‐VI semiconductors and their quantum well heterostructures.^[^ ^9^, ^10^, ^30^ ^]^

In an RSA experiment, carrier spin coherence is excited by a pulsed laser with repetition period *T*_R_, which equals to 13.2 ns in our experiments. After the laser pulse, the photoinduced carrier spin polarization precesses about the magnetic field applied in the Voigt geometry, while decaying with the spin dephasing time $T_{2}^{*}$. In the case, when the spin dephasing time is larger than the repetition period ($T_{2}^{*}>T_{R}$), the spin polarization does not fully decay up to the moment of the next pulse arrival. This next pulse can generate spin polarization which can interfere constructively with the decaying polarization. In this case, the total polarization is amplified. If on the other hand the two polarizations are in anti‐phase, destructive interference occurs minimizing the total polarization. Polarization accumulation occurs when the Larmor precession frequency is commensurate with the laser pulse repetition frequency, satisfying thereby the phase synchronization condition (PSC):

(2) $$\omega_{L}=n\omega_{R}=n2\pi /T_{R}\;.$$

**Figure 4:**
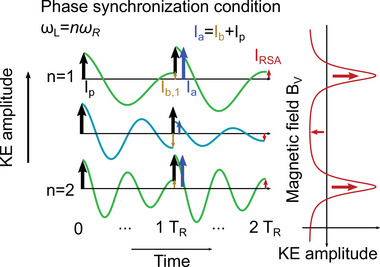
Resonant spin amplification effect. Sketch explaining how long‐lived spin dynamics lead to spin accumulation and RSA. Three KE dynamics traces are shown with different Larmor precession frequencies. Black arrows indicate the periodic generation of spin polarization with each pump pulse arrival (*I*_*p*_). Green curves give precession with the Larmor frequency ω_L_ being an integer number *n* of the pulse repetition frequency ω_R_, while the blue trace gives precession for ω_L_ = 1.5ω_R_. With arrival of the next pulse at 1*T*_R_, the remaining spin coherence (yellow arrows (*I*_*b*, 1_)) adds constructively or destructively for the integer and half‐integer relations cases, respectively, to the newly generated spin cohernce shown with the black arrows, resulting in the blue arrows (*I*_*a*_ = *I*_*b*_ + *I*_*p*_). The constructive case corresponds to the phase synchronization condition ω_L_ = *n*ω_R_. This sequence of additive pump pulses repeats quasi‐infinitely often leading to a strongly increased signal. The RSA signal (red curve) is obtained for continuously scanning the magnetic field, leading to a periodic match of the PSC condition. It is typically measured for small negative delays, i.e., shortly before the next pump pulse, marked by the red arrow in the dynamics.

Here, *n* is an integer. A schematic presentation of the spin accumulation in the RSA process is shown in **Figure** **4**.

A detailed theoretical consideration of the RSA effect can be found in ref. [30]. Here, we present the key equations from this paper. Considering indistinguishable spins, after the first pump pulse action, the spin dynamics oscillate with the Larmor frequency, decaying with the spin relaxation time τ_s_. To describe the process of spin accumulation, the amplitude of the spin signal (in our case, this is Kerr ellipticity amplitude $A_{\mathrm{KE}}^{\mathrm{RSA}}$) is calculated as sum of the spin dynamics over an infinite number of laser pulses:

(3) $$\begin{array}{ccc} A_{\mathrm{KE}}^{\mathrm{RSA}}\left(\omega_{L}\left(B\right),t\right) & = & \sum_{m=0}^{\infty }S_{0}\exp\left[-\left(t+mT_{R}\right)/\tau_{s}\right]\times \\ & & \cos\left[\omega_{L}\left(t+mT_{R}\right)\right]\;. \end{array}$$

Here *m* is the number of the pulse and *S*_0_ is the spin polarization induced by each pump pulse action. The summation over *m* gives:

(4) $$\begin{array}{c} A_{\mathrm{KE}}^{\mathrm{RSA}}\left(\omega_{L}\left(B\right),t\right)=\frac{S_{0}}{2}\exp\left[-t/\tau_{s}\right]\times \\ \frac{\exp\left[-T_{R}/\tau_{s}\right]\cos\left(\omega_{L}t\right)-\cos\left[\omega_{L}\left(t+T_{R}\right)\right]}{\cosh\left[T_{R}/\tau_{s}\right]-\cos\left(\omega_{L}T_{R}\right)}\;. \end{array}$$

**Figure 5:**
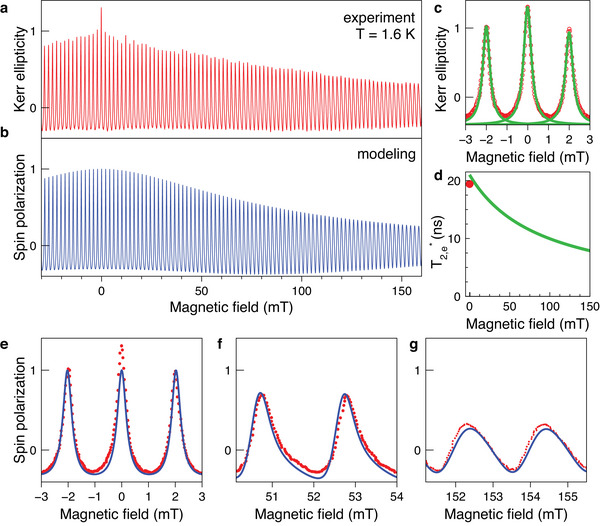
Resonant spin amplification of electrons in the MAPbI_3_ crystal measured at 1.637 eV photon energy. a) Magnetic field dependence of the Kerr ellipticity amplitude detected at the small negative delay of *t* = −50 ps. The magnetic field is applied in Voigt geometry. The pump power is 0.5 W cm^−2^ at *T* = 1.6 K. b) Simulated RSA signals using Equation (4) with the parameters *g*_e, 0_ = 2.676, Δ*g*_e_ = 0.006, and $\tau_{s}=T_{2,0}^{*}=21$ ns. c) The three RSA peaks around zero magnetic field (red dots give experimental data) and their fit by three Lorenzian functions (green lines) using Equation (5). d) Magnetic field dependence of the spin dephasing time $T_{2}^{*}$ (green line). We use Equation (6) with the parameters Δ*g*_e_ = 0.006, and $T_{2,0}^{*}=21$ ns. Red point is the value $T_{2,0}^{*}=19.4$ ns obtained from the Lorentzian fit in panel (c). e–g) Zoom into RSA peaks and their fit (blue line) with Equation (4).

Experimentally, for investigating the RSA effect it is favorable not to measure the time‐resolved dynamics, but fix the time delay at a small negative value and scan the magnetic field while detecting the KE amplitude. This allows one to perform measurements in weak magnetic fields, starting from zero field. Practically, the probe pulses are set in time to a value right before the arrival of the pump pulses. One describes this setting as a negative time delay of, e.g., *t* = −50 ps, which in fact corresponds to a time delay close to the laser repetition period, i.e. *T*_R_ − 50 ps. The amplitude of the spin signal is measured as function of the external magnetic field. The RSA signal calculated with Equation (4) shows an oscillating behavior with sharp resonances separated in magnetic field by ℏω_R_/μ_B_ *g*. The width of the resonances is determined by the spin relaxation time, see **Figure** **5**b.

Equation (4) can be simplified to a Lorentzian form at zero delay time *t* when the two conditions |ω_L_ *T*_R_ − 2π*n*| ≪ 1 and τ_s_ ≫ *T*_R_ are fulfilled:

(5) $$\begin{array}{c} A_{\mathrm{KE}}^{\mathrm{RSA}}\left(\omega_{L}\left(B\right)\right)∼\frac{1}{{\left(\omega_{L}T_{R}-2\pi n\right)}^{2}+{\left(T_{R}/\tau_{s}\right)}^{2}}. \end{array}$$

Equation (5) can be used for the evaluation of the spin relaxation time when the dispersion of the *g*‐factors is not significant and the RSA peaks remain sharp, which is fulfilled for the condition τ_s_ ≫ *T*_R_. To include the *g*‐factor dispersion in the model, Equation (4) should be averaged over the *g*‐factor distribution, see ref. [30] for details. However, this requires numerical calculations. An estimate of the *g*‐factor dispersion can be obtained by fitting a sequence of RSA peaks by Lorentzians and interpret the width as dephasing time.

An experimentally measured RSA signal is shown in Figure 5a. It exhibits periodic peaks with the period of ℏω_R_/μ_B_ *g* = 2 mT. By fitting the peak around *B*_V_ = 0 T using Equation (5), we obtain $\tau_{s}=T_{2,0}^{*}=19.4$ ns. The amplitude of the peaks decreases for higher magnetic fields due to the increasing significance of the *g*‐factor dispersion (Δ*g*), because the dispersion of Larmor frequencies (Δω_L_) increases with magnetic field, following Δ*g* = ℏΔω_L_/μ_B_ *B*_V_. To determine Δ*g*, we calculate an RSA signal by integrating Equation (4) using a Gaussian distribution of *g*‐factors. The magnetic field dependence of the RSA amplitude is shown in Figure 5b. The model parameters used are *g*_e,0_ = 2.676 (the median *g*‐factor in the distribution), Δ*g*_e_ = 0.006, and $\tau_{s}=T_{2,0}^{*}=21$ ns. The experimental and simulated curves show good agreement, which can be seen by comparing Figure 5a,b, as well as the details of the RSA peaks shown in Figure 5e–g. Note that the RSA peaks become broader and transform into a sinusoidal shape with increasing magnetic field due to the increasing contribution of the *g*‐factor dispersion. The *g*‐factor that we obtain from the modeling coincides with that of the electrons in MAPbI_3_. Therefore, the long living spin coherence can be uniquely assigned to localized electrons. It is worth to note that the measured spin dephasing time of $T_{\text{2,0,e}}^{*}=21$ ns is the longest reported so far for lead halide perovskite semiconductors, see our comments in the introduction on available literature. We attribute this finding to the high structural quality of the studied thin MAPbI_3_ single crystal, as well as to the favorite experimental conditions (*T* = 1.6 K and low laser density).

The spin dephasing time is decreasing with magnetic field due to the decreasing impact of the *g*‐factor dispersion and can be calculated as:^[^ ^10^ ^]^

(6) $$\frac{1}{T_{2}^{*}\left(B\right)}=\frac{1}{T_{2,0}^{*}}+\frac{\Delta g\mu_{B}B}{\hbar }.$$

Using Δ*g*_e_ = 0.006 and $T_{2,0}^{*}=21$ ns, we simulate this dependence and show it in Figure 5d (green line). The red dot gives the $T_{2,0}^{*}=19.4$ ns value estimated from the Lorentzian fit of a single peak, which agrees with the value obtained from the RSA curve modeling.

We emphasize that Δ*g*_e_ = 0.006 or Δ*g*_e_/*g*_e_ = 0.2% is a quite small value, evidencing the high homogeneity of the electron spin system. Let us compare it with the *g*‐factor difference for electrons excited on the high and low energy flank of the 1.5 ps pump pulse with finite spectral width. The electron *g*‐factor depends on the band gap energy (*E*_*g*_, which in our case can be considered as energy of the optical transition) as^[^ ^7^ ^]^

(7) $$\begin{array}{ccc} g_{e} & = & -\frac{2}{3}+\frac{4}{3}\frac{p^{2}}{m_{0}}\frac{1}{E_{g}}+\Delta g_{e}^{\prime }\;. \end{array}$$

Here, *p* is the interband matrix element of the momentum operator, *m*_0_ the electron rest mass, $\Delta g_{e}^{\prime }=-1$ the remote band contribution. In the lead halide perovskites, ℏ*p*/*m*_0_ = 6.8 eVÅ.^[^ ^7^ ^]^ For the energy range, covered by the pulse width of 1.5 meV we estimate a *g*‐factor difference of about 0.004, which is close to the Δ*g*_e_ = 0.006 extracted from the RSA curve.

### 2.4 Polarization Recovery

In Faraday geometry, the external magnetic field is applied parallel to the pump light wavevector and, therefore, parallel to the generated carrier spin polarization. These carriers possess no average spin projection perpendicular to the field and do not undergo Larmor spin precession in the external magnetic field. Therefore, one expects that their spin dynamics are controlled by the longitudinal spin relaxation time *T*_1_.

**Figure 6:**
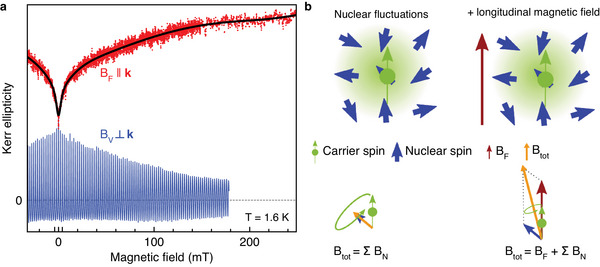
Polarization recovery. a) Kerr ellipticity measured at the small negative time delay *t* = −50 ps as function of the magnetic field scanned in Faraday geometry (red dots, PR curve) and in Voigt geometry (blue line, RSA). Black line is fit to the PR curve with a three‐Lorentzian function of Equation (8). The excitation density of the pump is 0.5 W cm^−2^ and of the probe is 0.35 W cm^−2^. b) Left: Sketch of carrier spin (green), interacting with several randomly oriented nuclear spins (blue) within the carrier localization volume, causing spin relaxation (mainly by dephasing in the nuclear fluctuation field). Right: Applying a magnetic field (brown arrow) along the spin polarization, can overcome the nuclear fluctuations stabilizing the carrier spin polarization.

However, at cryogenic temperatures, the nuclear spins play a significant role in the spin dynamics of localized carriers via the hyperfine interaction. It has been studied in detail for conventional III‐V and II‐VI semiconductors^[^ ^40^ ^]^ and also demonstrated for the lead halide perovskite crystals.^[^ ^13^, ^14^, ^34^ ^]^ Being localized, a carrier interacts with a finite number of nuclear spins, which have random spin orientations. The magnetic moment of the nuclear spin fluctuation acts on the carrier spin as an effective magnetic field, which is known as the nuclear Overhauser field. The nuclear fluctuations are also randomly oriented. Therefore, the carrier spin precesses around the perpendicular component of the nuclear Overhauser field, as sketched schematically in **Figure** **6**b. At zero external field, this precession provides carrier spin relaxation, but this mechanism can be suppressed in an external magnetic field with strength exceeding that of the nuclear fields. Detailed considerations of this effect with accounting for the perovskite specifics can be found in ref. [34]. Experimentally, it can be observed as polarization recovery (PR) effect, i.e., as an increases of the spin polarization signal in longitudinal magnetic field.^[^ ^40^ ^]^

We measure the PR in the studied MAPbI_3_ crystal by detecting the KE amplitude at a small negative delay of −50 ps. Its magnetic field dependence is shown by the red symbols in Figure 6a, where also the RSA signal is shown for comparison. The KE amplitude increases by a factor of 2 with the magnetic field increasing to *B*_F_ = 250 mT. One can see that several processes are involved in this dependence. In order to quantify them, we fit the PR magnetic field dependence with a function composed of several Lorentzians^[^ ^34^, ^41^ ^]^:

(8) $$A_{\mathrm{KE}}^{\mathrm{PR}}\left(B_{F}\right)=A_{\mathrm{KE},\mathrm{sat}}-\sum_{i}\frac{A_{i}}{1+{\left(\frac{B_{F}}{\delta_{\mathrm{PR},i}}\right)}^{2}}.$$

Here *A*_KE,sat_ is the saturation level of the KE amplitude at large magnetic fields and δ_PR, *i*_ is the characteristic half width at half maximum (HWHM) of the Lorentzians. A fit of the PR experimental data with Equation (8) is given by the black line in Figure 6a. It shows that the PR signal consists indeed of three components with: δ_PR,1_ = 3 mT, δ_PR,2_ = 21 mT, and δ_PR,3_ = 123 mT, and amplitudes *A*_KE,sat_ = 1, *A*_1_ = 0.16, *A*_2_ = 0.14, and *A*_3_ = 0.28.

By comparing these data with the recently published results for PR in FA_0.9_Cs_0.1_PbI_2.8_Br_0.2_, where we found for electrons δ_PR,e_ = 5 mT and for holes δ_PR,h_ = 19 mT,^[^ ^34^ ^]^ we assign the δ_PR,1_ = 3 mT to localized electrons and the δ_PR,2_ = 21 mT to localized holes in our sample. In the lead halide perovskites, the holes have a stronger hyperfine interaction with the nuclear spins compared to the electrons, which results in a broader PR curve. The origin of the process behind the broad signal with δ_PR,3_ = 123 mT needs further clarification. We suggest that it is related to mechanisms which are not related to nuclear spins, but provide a *T*_1_(*B*_F_) dependence for the carriers.

We show in Section 2.5 by spin inertia measurements that the longitudinal spin relaxation time *T*_1_ in MAPbI_3_ crystals is 20 ns at zero magnetic field and increases to 30 ns in *B*_F_ = 20 mT. Notably, this *T*_1_ time is longer than the pump laser repetition period *T*_R_ = 13.2 ns in our experiment. In this case the photogenerated spin polarization does not fully relax between the laser pulses and spin accumulation can occur. Its effect can be described by the following equation:

(9) $$A_{\mathrm{KE}}\left(t\right)=\sum_{m=0}^{\infty }S_{0}\exp[-\left(t+mT_{R}\right)/T_{1}]\;.$$

Further illustrations of the spin accumulation effect based on this modeling can be found in the Supporting Information.

### 2.5 Spin Inertia

To measure the longitudinal spin relaxation time *T*_1_, the spin inertia technique can be employed.^[^ ^42^, ^43^ ^]^ For that, the carrier spin polarization is driven by laser light, which can be pulsed or continuous‐wave, with periodically circular polarization modulated between σ^+^ and σ^−^ with a modulation frequency *f*_m_. During each period, the spin polarization induced during the previous period is repolarized toward the opposite orientation. The repolarization occurs within the characteristic spin lifetime *T*_s_, which depends on the longitudinal spin relaxation time *T*_1_, the carrier generation rate *G*, and the resident carrier concentration *n*_0_:

(10) $$\frac{1}{T_{s}}=\frac{1}{T_{1}}+\frac{G}{n_{0}}\;.$$

For a small generation rate, i.e., a weak excitation density, *T*_s_ ≈ *T*_1_.

**Figure 7:**
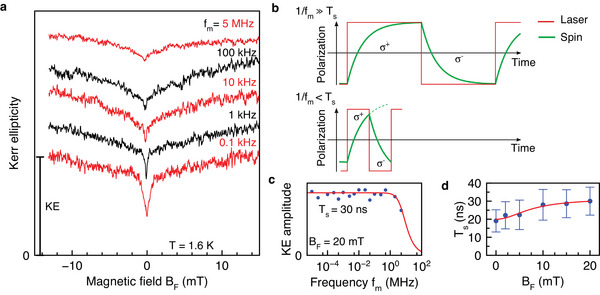
Spin inertia. a) Polarization recovery curves measured at the negative delay of −50 ps as function of magnetic field scanned in Faraday geometry for different helicity modulation frequencies. All dependences, except the one for 0.1 kHz, are shifted vertically for clarity. The excitation density of the pump is 0.5 W cm^−2^ and that of the probe is 0.35 W cm^−2^. b) Sketch of spin inertia. Upper diagram is for 1/*f*_m_ ≫ *T*_s_ and lower one for 1/*f*_m_ < *T*_s_. The spin polarization is driven by a train of alternatively σ^+^ and σ^−^ polarized periods (red line). The spin polarization (green line) shows inertia, following the excitation polaization change with some delay characterized with the time *T*_s_. c) KE amplitude in the saturation regime at *B*_F_ = 20 mT as function of helicity modulation frequency *f*_m_ (blue symbols). Red line is a fit with Equation (11) giving *T*_s_ = 30 ns. d) Magnetic field dependence of *T*_s_ (blue symbols) where the red line is a guide for the eye.

One can see in the diagrams of **Figure** **7**b that if the polarization modulation period greatly exceeds *T*_s_ (i.e., 1/*f*_m_ ≫ *T*_s_) the spin polarization reaches its maximal saturation value. However, for faster modulation (1/*f*_m_ < *T*_s_) the maximal amplitude is not reached. Therefore, experimentally *T*_s_ can be evaluated by measuring the KE amplitude as function of *f*_m_ and fitting it with the form^[^ ^42^ ^]^

(11) $$A_{\mathrm{KE}}\left(f_{m}\right)\propto \frac{1}{\sqrt{1+{\left(2\pi f_{m}T_{s}\right)}^{2}}}\;.$$

The measurements can be performed at various magnetic fields applied in the Faraday geometry to obtain the *T*_s_(*B*_F_) dependence.

For the studied MAPbI_3_ sample, the measurements are performed at the small negative delay of −50 ps, where the electron spin polarization mainly contributes to the spin dynamics. Examples of PR curves measured for polarization modulation frequencies in the range from 0.1 kHz up to 5 MHz are shown in Figure 7a. Note that these PR curves are shifted vertically for clarity. The KE amplitude measured at *B*_F_ = 20 mT as function of *f*_m_ is shown in Figure 7c. There is a clear decrease of the KE amplitude for the highest frequency of 5 MHz. A fit with Equation (11) yields *T*_s_ = 30 ns, which exceeds *T*_R_ = 13.2 ns, confirming the spin accumulation regime for the electrons. Note that we use a relatively low excitation density in this experiment, so that *T*_s_ ≈ *T*_1_ and we can conclude that *T*_1_ ⩾ 30 ns.

The magnetic field dependence of *T*_s_(*B*_F_) in the field range up to 20 mT is given in Figure 7d. *T*_s_(*B*_F_) increases from 20 ns at zero field up to 30 ns at 20 mT, in line with the PR dependence shown in Figure 6a.

### 2.6 Dynamic Nuclear Polarization

The hyperfine interaction with the nuclear spins plays the dominant role in the spin relaxation of electrons and holes in lead halide perovskite semiconductors at cryogenic temperatures.^[^ ^13^, ^34^, ^44^ ^]^ The nuclear spins have random orientations and provide efficient spin relaxation of the carriers,^[^ ^45^ ^]^ while the spin relaxation of the nuclear spins takes a long time reaching seconds in perovskites. This can be used to preserve the spin orientation of carriers for a long time and, therefore, enhance spin accumulation effects. Also, adjustment of Larmor precession frequencies by the nuclear frequency focusing effect in the spin mode‐locking regime^[^ ^46^ ^]^ can be used to modify the coherent spin dynamics of carriers.^[^ ^31^ ^]^

Spin polarized carriers can transfer their polarization to the nuclear spin system, inducing a dynamic nuclear polarization (DNP). In turn, the polarized nuclei act on the carrier spins via the nuclear Overhauser field, which can be considered as effective magnetic field inducing carrier Zeeman splitting. The nuclear Overhauser field in combination with the external magnetic field changes the Larmor precession frequency of the carriers and, therefore, can be detected in time‐resolved KE experiments.^[^ ^13^ ^]^ This experiment requires a tilted geometry of the magnetic field, where the external field component perpendicular to the carrier spin polarization (i.e., perpendicular to the pump beam *k*‐vector) is responsible for the Larmor precession, while the external field component parallel to the carrier spin polarization provides the spin transfer for achieving DNP. The induced DNP reads

(12) $$\left\langle I\right\rangle =l\frac{4I(I+1)}{3}\frac{B(B\cdot \left\langle S_{\text{e(h)}}\right\rangle )}{B^{2}}\;.$$

Here l is a leakage factor, *I* is the nuclear spin, **S**_e(h)_ is the steady‐state polarization of the carriers induced by optical orientation, for details see refs. [13, 47].

**Figure 8:**
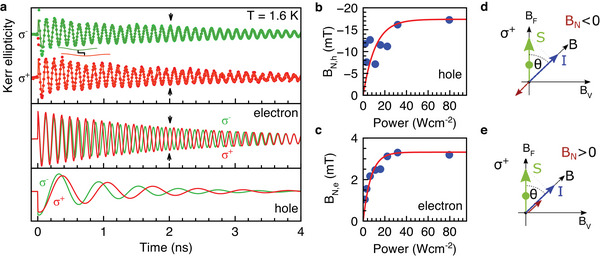
Dynamic nuclear polarization. a) KE dynamics measured with σ^+^ (red) or σ^−^ (green) pump polarization at $B=\sqrt{\left(B_{F}^{2}+B_{V}^{2}\right)}=0.2$ T, tilted by the angle θ = 60° from the Faraday geometry. The pump power is 32 W cm^−2^. The dynamics are fitted with Equation (16) from which the electron and hole components are extracted and given below with corresponding colors. Note that the σ^−^ data are multiplied by −1, to have the same signal sign at *t* = 0 as the σ^+^ data. DNP results in the emergence of the nuclear Overhauser field *B*_N_ acting on the carriers and shifting their Larmor precession frequency. The considerable difference in the electron oscillation frequency is highlighted by the arrows at 2 ns, showing opposite amplitudes. For the holes, it is evidenced as shift of an overtone‐like modulation of the curves, as indicated at 0.5 ns. b,c) Overhauser field *B*_N_ for the holes and the electrons, calculated according to Equation (13). d,e) Sketches illustrating the DNP mechanism mediated by the holes and the electrons. Note that the difference in the Overhauser field sign for electrons and holes comes from the opposite signs of their *g*‐factors.

The experimental demonstration of the DNP and its detection via time‐resolved KE are shown in **Figure** **8**a. Here the magnetic field of 0.2 T is tilted by the angle of θ = 60° from the Faraday geometry. The spin dynamics are measured for constant pump helicity, either σ^+^ (red symbols and line) or σ^−^ (green symbols and line). For better comparison, we invert the phase of the σ^−^ pumped dynamics by multiplying them by −1. We fit the KE dynamics with Equation (16) and plot the electron and hole components in the lower panels of Figure 8a. One can see, that the change of the pump helicity sign has an effect on the Larmor precession frequency both for electrons and for holes. This directly demonstrates the presence of a nuclear Overhauser field, which for the σ^+^‐polarized pump either adds to the external magnetic field, as in case of the electrons which Larmor frequency becomes higher, or subtract from it as in case of the holes, which frequency reduces. This difference arises from the opposite signs of the electron and hole *g*‐factors in MAPbI_3_. The diagrams in Figure 8d,e give more details of the DNP process involving the holes and the electrons.

The nuclear Overhauser field can be evaluated from the difference in Larmor precession frequencies measured for σ^+^ and σ^−^ polarized pumps:

(13) $$B_{\text{N,e(h)}}=\frac{\hbar (\omega_{+}-\omega_{-})}{2g_{\text{e(h)}}\mu_{B}}.$$

where ω_±_ are the Larmor precession frequencies for a σ^±^ pump. The evaluated Overhauser fields using *g*_h_ = −0.44 and *g*_e_ = +2.76 are plotted as function of the pump power in Figures 8b,c. Both for holes and electrons an initial increase converts to saturation. The saturation is due to the interplay between a higher carrier spin polarization with increasing pump power and an acceleration of the carrier spin relaxation, see Figure 3. Note that the Overhauser field is considerably stronger for holes than for electrons, which is common for the lead halide perovskites.^[^ ^13^ ^]^ This difference is originated from the band structure of the perovskite semiconductors, where the lead orbitals contribute dominantly to the electronic states forming the bandgap, i.e., both the bottom of conduction band and the top of valence band. The valence band are formed by the *s*‐type of the lead orbitals having large overlap with hole wavefunction and, therefore, strong hyperfine interaction. The conduction band is contributed by the *p*‐type of the lead orbitals with an admixture of the *s*‐type orbitals of iodine halogen and this provides relatively weak hyperfine interaction for electrons.

### 2.7 Impact of Lattice Temperature

**Figure 9:**
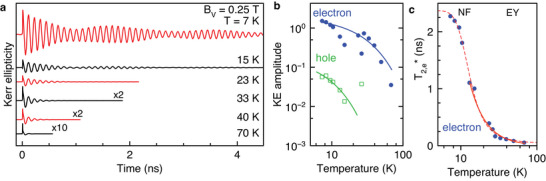
Temperature dependence of spin dynamics. a) KE dynamics at different temperatures. The dynamics are fitted with Equation (16) and the evaluated parameters are shown in panels (b,c). b) Temperature dependence of the amplitudes of the carrier spin components (dots). Lines are for the eye. Note the double logarithmic scale. c) Temperature dependence of the electron spin dephasing time (symbols). Fits are shown using Equation (15) (solid line) for *T* ⩾ 10 K and using an Arrhenius form according to Equation (14) (dashed line). Excitation density of pump is 3.2 W cm^−2^ and of probe 0.8 W cm^−2^.

The carrier spin dynamics depend sensitively on the lattice temperature. A set of KE dynamics in the temperature range from 7 to 70 K is shown in **Figure** **9**a. With increasing lattice temperature the signal amplitude decreases rapidly and the spin dephasing accelerates. The dynamics are analyzed by fits with Equation (16). The resulting temperature dependences of the KE amplitude and the electron spin dephasing time ($T_{\text{2,e}}^{*}$) are presented in Figures 9b,c. Note that above 23 K the pump photon energy is tuned with temperature according to the law *E*_λ_/*T* = +0.5 meVK^−1^ with an offset of 1.630 eV, in order to accordingly achieve maximum signal.

The KE amplitude decreases strongly for growing temperature, see Figure 9b. The spin dephasing time can be evaluated only for the electrons, as the accuracy is too low for the holes. A phenomenological Arrhenius‐like activation function can describe well its temperature dependence:

(14) $$\frac{1}{T_{2}^{*}\left(T\right)}=\frac{1}{T_{2,0}^{*}}+w\exp\left(-\frac{E_{A}}{k_{B}T}\right)\;.$$

Here *E*_*A*_ is the activation energy, *k*_B_ is the Boltzmann constant, and $T_{2,0}^{*}$ is the temperature independent spin dephasing time. A fit shown by the dashed line gives *w* = 0.032 nsK^−1^, $T_{2,0}^{*}=2.4$ ns, and *E*_*A*_ = 5 meV, in agreement with the lowest phonon modes in MAPbI_3_.^[^ ^48^ ^]^ Qualitatively, the strong shortening of the $T_{\text{2,e}}^{*}$ time with increasing temperature can be explained by electron delocalization and the involvement of other spin relaxation mechanisms relevant for free carriers.

As the Dyakonov–Perel spin relaxation mechanism is inefficient in bulk lead halide perovskites,^[^ ^33^, ^37^ ^]^ the most relevant candidate is the Elliott–Yafet (EY) mechanism. It has the following temperature dependence:^[^ ^47^ ^]^

(15) $$\frac{1}{\tau_{s}^{EY}}=A_{\mathrm{EY}}\eta^{2}{\left(\frac{1-\eta /2}{1-\eta /3}\right)}^{2}{\left(\frac{k_{B}T}{E_{g}}\right)}^{2}\frac{1}{\tau_{P}}\;.$$

Here $\tau_{s}^{EY}$ is the spin relaxation time for the EY mechanism, *A*_EY_ is a constant, η = Δ_SO_/(*E*_*g*_ + Δ_SO_) with Δ_SO_ ≈ 1.5 eV being the spin‐orbit coupling, *E*_*g*_ is the band gap, and 1/τ_*P*_ is the momentum scattering rate. Apparently, for a fit *A*_EY_ and 1/τ_*P*_ are interdependent, thus we set *A*_EY_ = 1 and get τ_*P*_ = 9.5 ns. The fit shown by the solid line in Figure 9c is made for *T* ⩾ 10 K, where the electrons are delocalized and can be treated as free.

For low temperatures the spin relaxation is dominated by the hyperfine interaction limiting the relaxation times. The general picture of the contributing relaxation mechanisms is complex and depends strongly on the degree of localization of the carriers, which will be subject to further investigations.

## 3 Discussion

To summarize the spin parameters obtained for MAPbI_3_ thin crystals in this study, we collect them in **Table** **1**. The data for bulk MAPbI_3_ crystals from ref. [14] are given for comparison. The longitudinal spin relaxation times *T*_1_ are comparable, lying in the range of about 30 ns. The hole spin dephasing times $T_{\text{2,h}}^{*}$ also do not differ strongly amounting to a few ns. However, the electron spin dephasing times $T_{\text{2,e}}^{*}$ differ strongly. $T_{\text{2,e}}^{*}$ is only 0.4 ns in bulk crystals and reaches a record value of 21 ns for the thin crystal. Note that the short electron spin dephasing time of 0.4 ns reported in ref. [14] is not an inherent feature of MAPbI_3_ bulk crystals, as recently $T_{\text{2,e}}^{*}=11$ ns was reported for MAPbI_3_ bulk crystals.^[^ ^15^ ^]^ The small dispersion of the carrier *g*‐factors Δ*g* align with the high structural quality of bulk and thin crystals, confirmed by the narrow spectral lines of excitons in low‐temperature reflectivity and PL. The maximum observed nuclear Overhauser field acting on the holes of −17.2 mT in the thin crystals is larger but still close to the −10.8 mT found in bulk crystals.

**Table 1:** Spin dynamics parameters of electrons and holes in bulk and thin crystals of MAPbI_3_ at *T* = 1.6 K. The bulk values are taken from ref. [14].

	bulk		thin crystal	
	hole	electron	hole	electron
*A*_h_/*A*_e_	≈ 1/1		≈ 1/5	
*T*_1_ (ns)	⩽37	⩽37	−	30
$T_{2,max}^{*}$ (ns)	2.7	0.4	0.8	21
Δ*g*	0.003 − 0.008	0.03 − 0.04	−	0.006
DNP_max_ (mT)	−10.8	+1.7	−17.2	+3.3

Interestingly, the hole spin signal is much weaker in thin crystals as compared to bulk, which might be related to a reduced hole abundance in thin single crystals.^[^ ^39^ ^]^ This in turn is an important piece of information to unravel the origin of the simultaneous hole and electron signals in perovskite crystals.

In respect of the spin relaxation times the lead halide perovskites are approaching or comparable with common semiconductors. To illustrate that one can use the data from Table 1 for MAPbI_3_ crystals and further results for perovskite crystals summarized in ref. [16]. Let us compare them with the longest spin relaxation times reached at cryogenics temperatures (typically 1.6 K) in several semiconductors with zinc blend crystal structure. We take here for comparison data for electrons in bulk materials, where the electrons are often localized on donors. Dependence of *T*_1_ time on doping concentration in n‐type GaAs can be found in ref. [49], the longest time here at moderate concentrations reaches 180 ns. *T*_1_ = 270 ns was reported for n‐type GaAs in ref. [50]. The spin dephasing times $T_{2}^{*}$ measured by RSA are of 15 ns in ZnO,^[^ ^51^ ^]^ 30 ns in ZnSe,^[^ ^52^ ^]^ 130 ns in GaAs,^[^ ^25^ ^]^ and by extended pump‐probe RSA of 230 ns in GaAs.^[^ ^50^ ^]^ The spin dephasing times addressed by spin noise techniques are of 23 ns in ZnO^[^ ^53^ ^]^ and about 1 µs in GaAs.^[^ ^54^ ^]^ Note, that hole spin relaxation times in zinc blend semiconductors are considerably shorter than the electron times due to complex band structure with heavy‐ and light‐hole states and strong spin‐orbit interaction.

## 4 Conclusion

To summarize, investigation of the long‐lived carrier spin dynamics in thin MAPbI_3_ single crystals allows us to demonstrate spin accumulation effects with conventional pulsed lasers operating at 76 MHz repetition rate. The resonant spin amplification, spin inertia, and polarization recovery techniques, as established methods for studying the spin physics in conventional III‐V and II‐VI semiconductors, are suitable tools also for the lead halide perovskite semiconductors. A long longitudinal spin relaxation of 30 ns and a electron spin dephasing time of 21 ns are measured in the MAPbI_3_ thin crystals. We are convinced that similarly long times can be reached in high quality lead halide perovskite crystals with other chemical compositions. We propose that the applied magneto‐optical techniques based on spin accumulation will significantly advance the understanding of the spin dynamics in perovskite semiconductors, particularly in experiments involving laser systems with GHz repetition rates. All these results position the lead halide perovskites as promising functional materials for spintronics and information technologies, which potential can be greatly extended by means of charge carrier confinement in perovskite nanocrystals and 2D materials. Already present results on the spin properties of the lead halide nanocrystals are promising for that,^[^ ^18^, ^31^, ^45^, ^55^ ^]^ especially in view of demonstration spin coherence and all‐optical spin manipulation at a room temperature.^[^ ^56^, ^57^, ^58^ ^]^

## 5 Experimental Section

### Samples

The investigated MAPbI_3_ thin single crystals were synthesized utilizing PI_2_ and MAI perovskite precursors. These precursors were introduced between two polytetrafluoroethylene‐coated glass substrates and subjected to a gradual thermal treatment, reaching a temperature of 120°C. A precursor molar ratio of PI_2_:MAI of 4:7 was employed. The thin single crystals exhibit a square morphology within the (001) crystallographic plane, with a thickness of approximately 20 µm. At ambient temperature, the MAPbI_3_ thin crystals have a tetragonal crystal structure, characterized by an out‐of‐plane tetragonal [001] axis. At cryogenic temperatures below 160 K the crystal structure changes to orthorhombic. The sample code is M2‐5. The sample has a size of 2 × 2 × 0.02 mm. An X‐ray diffraction (XRD) analysis performed at room temperature confirmed the crystallographic orientation of the sample surface normal which is collinear to the light wave vector, **k**∥[001] (c‐axis).

### Magneto‐Optical Measurements

For magneto‐optical experiments the sample is placed in a cryostat allowing a temperature variable from 1.6 K up to 300 K. For *T* = 1.6 K the sample is immersed in superfluid helium, while for temperatures in the range from 4.2 to 300 K the sample is held in helium cooling gas. The cryostat is equipped with a vector magnet composed of three superconducting split coils oriented orthogonal to each other. This allows us to apply magnetic fields up to 3 T in any direction. We use a Cartesian coordinate system with the z‐axis collinear to the sample surface normal (i.e., to the c‐axis) and to the light wave vector **k**. The magnetic field collinear to the z‐axis corresponds to the Faraday geometry (**B**_F_∥**k**). The magnetic field oriented in the plane perpendicular to **k** corresponds to the Voigt geometry (**B**_V_⊥**k**). Note that the 3D vector magnet allows also for a precise compensation of the residual fields.

### Photoluminescence and Reflectivity

Time‐resolved photoluminescence (PL) is excited by a 200 fs‐pulsed Ti:Sa laser emitting at the photon energy of 1.771 eV (700 nm). For detection we use a streak camera attached to an 0.5‐m spectrometer, providing a time‐resolution of about 20 ps. For the reflectivity measurements a halogen lamp is used and the reflected light is coupled via a fiber into an 0.5 m spectrometer, equipped with a Peltier‐cooled silicon charge‐coupled device (CCD).

### Time‐Resolved Kerr Ellipticity

The coherent spin dynamics of electrons and holes interacting with the nuclear spins are measured by a degenerate pump‐probe technique, where the pump and the probe have the same photon energy.^[^ ^10^ ^]^ A titanium‐sapphire laser generates 1.5 ps long pulses with a spectral width of about 1 nm (about 1.5 meV) at a pulse repetition rate of 76 MHz (repetition period *T*_R_ = 13.2 ns). The output photon energy is tuned to be about at the exciton resonance in the reflectivity spectrum to achieve maximum Kerr ellipticity signal at 1.637 eV (757.3 nm). The laser output is split into the pump and probe beams. The probe pulses are delayed relative to the pump pulses by a double‐pass mechanical delay line with one meter length. The pump and probe beams are modulated using a photo‐elastic modulator (PEM) for the probe and an electro‐optical modulator (EOM) for the pump. The probe beam is always linearly polarized with the amplitude modulated at the frequency of 84 kHz, with the PEM set to the λ/2 mode and combined with a Glan prism. The pump and probe beams are focussed on the sample, with pump spot diameter being 200 µm and the probe spot size being slightly smaller.

The pump beam is either helicity modulated between σ^+^ and σ^−^ circular polarizations, or amplitude modulated with fixed helicity, either σ^+^ or σ^−^, in the frequency range from 0 to 5 MHz. In all cases *f*_m_ refers to the helicity modulation frequency. Amplitude modulation can in effect be considered as 0 Hz helicity modulation, as the signal is independent of the bare amplitude modulation frequency. In the experiment, typically 20 Hz to 100 kHz helicity and amplitude modulation frequencies are used. The polarization of the reflected probe beam is analyzed with respect to the rotation of its elliptical polarization (Kerr ellipticity) using a balanced photodiode with a lock‐in technique. The time‐resolved Kerr ellipticity dynamics are analyzed via fits with the equation 

(16) $$A_{\mathrm{KE}}\left(t\right)=\sum_{i=e,h}A_{i}\cos\left(\omega_{L,i}t\right)\exp\left(-t/T_{2,i}^{*}\right).$$

Here *i* = *e*, *h* labels the electron and hole components, *A*_*i*_ is the component specific amplitude, ω_L, *i*_ is the Larmor precession frequency, $T_{2,i}^{*}$ is the spin dephasing time of the carrier spin ensemble. For simplicity the *i* index is not used, if not explicitly needed. Note, that $T_{2}^{*}\leq T_{2}$, where *T*_2_ is the spin coherence time of the individual carriers.

## Conflict of Interest

The authors declare no conflict of interest.

## Supporting information

Supporting Information

## Data Availability

The data that support the findings of this study are available from the corresponding author upon reasonable request.
