# Supplementary material for: Resonant Spin Amplification and Accumulation in MAPbI3 Single Crystals
Source: Adv Sci (Weinh). 2025 May 7;12(27):2502735. doi: 10.1002/advs.202502735 (PMC12279217; doi:10.1002/advs.202502735)
Supplement: Supplementary file 1 — Supporting Information [file ADVS-12-2502735-s001.pdf]

ADVANCED SCIENCE
Open Access

Supporting Information

for *Adv. Sci.*, DOI 10.1002/advs.202502735

Resonant Spin Amplification and Accumulation in $MAPbI_3$ Single Crystals

Erik Kirstein, Dmitri R. Yakovlev*, Evgeny A. Zhukov, Nataliia E. Kopteva, Bekir Turedi, Maksym V. Kovalenko and Manfred Bayer*

Resonant spin amplification and accumulation in $MAPbI_3$ single crystals

Erik Kirstein[1], Dmitri R. Yakovlev[1], Evgeny A. Zhukov[1], Nataliia E. Kopteva[1], Bekir Turedi[2,3], Maksym V. Kovalenko[2,3], Manfred Bayer[1,4]
[1]*Experimentelle Physik 2, Technische Universität Dortmund, 44227 Dortmund, Germany*
[2]*Department of Chemistry and Applied Biosciences, Laboratory of Inorganic Chemistry, ETH Zürich, 8093 Zürich, Switzerland*
[3]*Department of Advanced Materials and Surfaces, Laboratory for Thin Films and Photovoltaics, Empa - Swiss Federal Laboratories for Materials Science and Technology, 8600 Dübendorf, Switzerland and*
[4]*Research Center FEMS, Technische Universität Dortmund, 44227 Dortmund, Germany*

I. SPIN ACCUMULATION IN PR CURVES

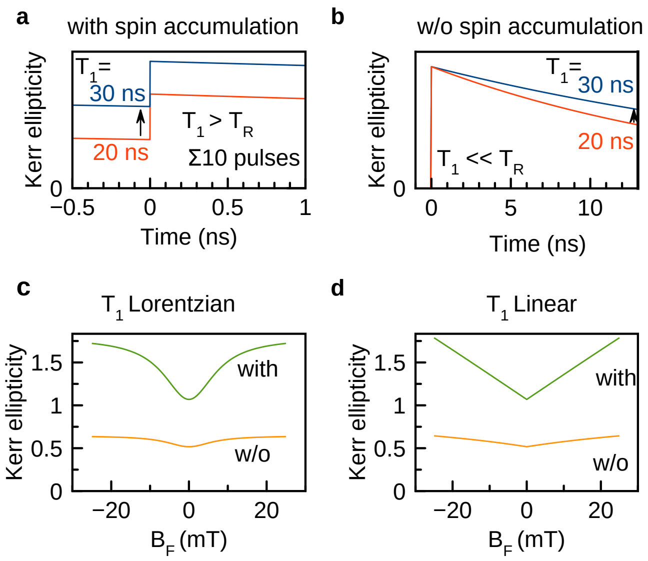

FIG. S1. Spin accumulation in PR curves. The non-linear rise of the PR curves with increasing magnetic field due to the increase of T_1 can be motivated by the time dynamics. (a) For $T_1 > T_R$, simulated here for a sequence of 10 pulses, at small negative delay ($t' = t - T_R = -50$ ps) the rise of the KE amplitude is strongly amplified. The black arrows in (a,b) mark similar times of ≈ 13 ns. (b) In case of single pulse excitation, e.g. if the inverse of the laser repetition rate $1/T_R$ is much longer than the spin dynamics time ($T_1 \ll T_R$), no spin accumulation takes place and the difference in KE amplitude at $t \approx 13.2$ ns-50 ps, for either $T_1 = 20$ ns or $T_1 = 30$ ns, is small. (c) KE amplitude at $t \approx 13$ ns for the cases with and without spin accumulation considered (i.e., the cases of long and short T_R) as shown in (a,b) with Lorentzian $T_1(B) \propto 30\,\text{ns} - \frac{10\,\text{ns}}{1+(B/8\,mT)^2}$. (d) similar to (c) with linear $T_1(B) = 0.4\,\text{ns/mT} \cdot B + 20\,\text{ns}$.

To illustrate the spin accumulation effect observed in the PR curves, we calculate the spin dynamics according to $\text{KE}(t) = \sum_{j=1}^{j_{\max}} \exp\left[-(t + jT_R)/T_1\right]$ with $j_{\max} = 1$ for $T_1 \ll T_R$ and $j_{\max} = 10$ for $T_1 > T_R$, representing the cases without (w/o) and with spin accumulation. In order to be close to the experimental results, we compare cases for T_1 within the range of $20 - 30$ ns, Figure S1a,b. Notably, without spin accumulation the rise of amplitude for increasing T_1 by a factor of 1.5 from 20 ns to 30 ns results in an increase of the KE amplitude by a factor of 1.24, while with spin accumulation the factor is 1.67 and the overall KE amplitude is larger.

Calculations for the regime $T_1 > T_R$ are shown in Figure S1a, where the modeling is made for the two times $T_1 = 20$ ns and 30 ns. The strong spin accumulation effect is evidenced by a finite KE amplitude at negative time delays. And the effect increases for longer T_1 as expected. The spin accumulation is also seen through the larger amplitude at positive delays for $T_1 = 30$ ns compared to the one for 20 ns. Figure S1b shows the modeling for the regime with $T_1 \ll T_R$, where spin accumulation is not expected. The same $T_1 = 20$ ns and 30 ns are taken as parameters together with $T_R = 1000$ ns. One can see here that the KE amplitude is zero at negative delays as the spin polarization fully relaxes between the pulses. The KE signal increase at long time delays, in the comparison of the 20 ns and 30 ns acases, is smaller than in the case with spin accumulation.

Next we simulated different dependences of $T_1(B)$, Figure S1c,d. With a Lorentzian dependence of T_1 the resulting PR curve is also Lorentzian-shaped and has the same width as the $T_1(B)$ Lorentzian. Again, one finds a much stronger contrast of the zero field to elevated field KE signal in the case where spin accumulation is considered. For the case without spin accumulation the dependence is rather flat. For a linear dependence of $T_1(B)$, in the case with spin accumulation the PR curve has a linear shape. Interestingly in the case without spin accumulation the rise of the PR curve rather follows a tangent hyperbolic shape, which might fit better to the most narrow PR peak as a Lorentzian and thus might indicate a component with rather short T_1 and linear $T_1(B)$. Note that in all cases the curves have been symmetrized by usage of absolute B values.
